# An Improved Automated High-Throughput Efficient Microplate Reader for Rapid Colorimetric Biosensing

**DOI:** 10.3390/bios12050284

**Published:** 2022-04-28

**Authors:** Jinhu Yang, Yue Wu, Hao Wang, Wenjian Yang, Zhongyuan Xu, Dong Liu, Hui-Jiuan Chen, Diming Zhang

**Affiliations:** 1 State Key Laboratory of Optoelectronic Materials and Technologies, Guangdong Province Key Laboratory of Display Material and Technology, School of Electronics and Information Technology, State Key Laboratory of Ophthalmology, Zhongshan Ophthalmic Center, Guangdong Provincial Key Laboratory of Ophthalmology and Visual Science, Sun Yat-sen University, Guangzhou 510006, China; yangjh93@mail2.sysu.edu.cn (J.Y.); wangh595@mail2.sysu.edu.cn (H.W.); 2 Research Center for Intelligent Sensing Systems, Zhejiang Laboratory, Hangzhou 311100, China; wuyue@zhejianglab.edu.cn (Y.W.); yangwenjian@zhejianglab.edu.cn (W.Y.); xuzy@zhejianglab.edu.cn (Z.X.)

**Keywords:** efficient microplate reader, high-throughput, full spectral absorbance, reagent screening

## Abstract

A high-throughput instrument to measure the full spectral properties of biochemical agents is necessary for fast screening in fields such as medical tests, environmental monitoring, and food analysis. However, this need has currently not been fully met by the commercial microplate reader (CMR). In this study, we have developed an automated high-throughput efficient microplate reader (AHTEMR) platform by combining a spectrometer and high-precision ball screw two-dimensional motion slide together, for high-throughput and full-spectrum-required biochemical assays. A two-dimensional slide working on a ball screw was driven by a stepper motor with a custom-designed master control circuit and used as a motion system of the AHTEMR platform to achieve precise positioning and fast movement of the microplate during measurements. A compact spectrometer was coupled with an in-house designed optical pathway system and used to achieve rapid capture of the full spectral properties of biochemical agents. In a performance test, the AHTEMR platform successfully measured the full spectral absorbance of bovine serum albumin (BSA) and glucose solution in multiple wells of the microplate within several minutes and presented the real-time full spectral absorbance of BSA and glucose solution. Compared with the CMR, the AHTEMR is 79 times faster in full-spectrum measurements and 2.38 times more sensitive at the optimal wavelength of 562 nm. The rapid measurement also demonstrated the great capacity of the AHTEMR platform for screening out the best colorimetric wavelengths for tests of BSA and glucose development, which will provide a promising approach to achieving high-throughput and full-spectrum-required biochemical assays.

## 1. Introduction

A microplate reader is a useful instrument for high-throughput measurements due to its non-contacted optical measurement without containment towards a photoelectric detector or biochemical samples. Up to now, the microplate reader has achieved various biochemical quantitative assays by counting photons emitted from biochemical samples at selective wavelengths. In medical tests, the microplate reader works with enzyme-linked immunosorbent assay (ELISA) as a high-throughput diagnostic tool to detect the presence of biomarkers in blood or urine [1,2,3,4]. In environmental monitoring, the microplate reader is always used to detect and analyze the pollutants in a liquid environmental sample to evaluate water quality [5,6,7]. In food analysis, the microplate reader plays an important role in the rapid and high-throughput quantification of amino acids, sweet components, and vitamins [8]. Overall, the microplate reader is an essential tool for high-throughput biochemical measurements in fields such as medical tests, environmental monitoring, and food analysis.

The basic working principle of the microplate reader is to use the photoelectric colorimeter or spectrophotometer to measure the energy difference in light before and after passing through the test substance [9,10,11,12,13]. The energy difference in light caused by the absorption of test the substance is usually linearly related to the concentration of the test substance. Hence, the microplate reader can quantify the concentration of the test substance via the optical measurement of light absorption at selective wavelengths. The widespread applications of the microplate reader in biochemical measurements have demonstrated several advantages such as high-throughput in one measurement, small volume requirement for the test substance, and highly automated data recording [14,15,16]. However, the conventional microplate reader has two limitations. Firstly, the microplate reader is not fast enough for spectral measurements and it requires several hours or even more time in measuring the full spectrum of the test substance, especially in measuring multiple wells or when repeated trials are required [16,17,18]. During this long measurement process, biological and chemical materials in the microplates may undergo significant changes in their optical properties (e.g., color and absorption) because of the prolonged exposure of the material to the air. Accurate quantification of the test substance, therefore, cannot be obtained in the full-spectrum measurement by the microplate reader. Secondly, the microplate reader cannot obtain a real-time spectrogram and it only provides numerical information about the absorbance of selective wavelengths during the measurement process [19,20,21]. Researchers have to plot spectrograms via extra software and draw conclusions after collecting the whole numerical information. Instant judgment is restricted in some cases with real-time monitoring, such as in water quality detection and food safety tests.

Microspectrometers have a great capacity for measuring the full spectrum at high speed and presenting a real-time spectrogram, which partially compensates for the shortcomings of a conventional microplate reader [10,22,23]. Moreover, microspectrometers are low in cost, compact in size, and light in weight, so are convenient for portable measurement in terms of large-scale trials outside the laboratory [24,25,26]. However, microspectrometers require a fixed light source and a photoelectric detector to measure the optical spectrum of test substances in the individual cuvette, and manual replacement of the cuvette is necessary to measure the spectrums of different test substances, which lowers the throughput of the microspectrometer in a single channel and can induce extra unpredictable measurement errors between different tests [27,28,29]. The low throughput and automation restrict applications of microspectrometers in biochemical assays that require large-scale screening; although, a microspectrometer has the capacity of measuring the full spectrum of biochemical components rapidly. Therefore, it would be promising for biochemical assays to build a platform that can integrate the microspectrometer into the conventional microplate reader for high-throughput and full-spectral measurements of biochemical substances.

**Figure 1:**
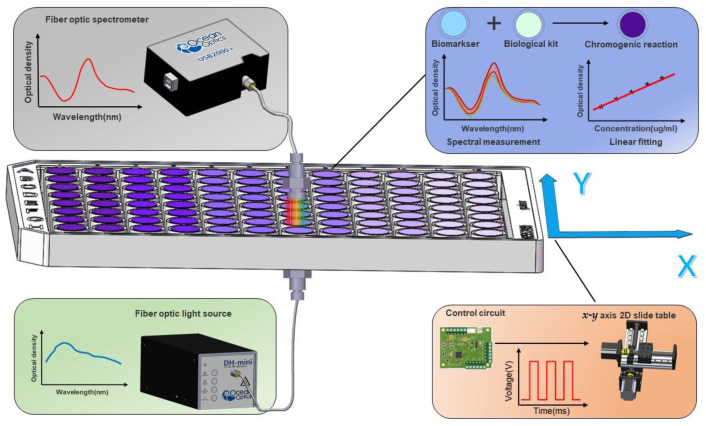
Mechanism of the automated high-throughput efficient microplate reader (AHTEMR) biosensing system. The AHTEMR consists of a micro fiber optic spectrometer, a light source, an *x*-*y* axis 2D Slide table, and a computer with LabVIEW software. Based on the self-designed master circuit and PWM signal, the *x*-*y* axis 2D slide table carrying the micro-hole plate can be precisely controlled to complete an accurate displacement in the plane.

In this study, we have developed an automated high-throughput efficient microplate reader (AHTEMR) platform by engineering a combination of a spectrometer and a high-precision ball screw two-dimensional motion slide for high-throughput and full-spectrum-required biochemical assays (Figure 1). The AHTEMR platform consists of three parts: the mechanical part, the electronic hardware part, and the computer software part. In the mechanical part, a high-precision ball screw two-dimensional motion slide (positioning accuracy of 0.03 mm) is used to achieve a precise two-dimensional movement of the instrument in the *x* and *y* directions. A custom-designed multi-well plate carrier and a vertical optical path holder are fabricated together to couple the optical path of the spectrometer and the testing well of the multi-well plate. In the electronic hardware part, a custom-designed master control circuit is built to receive commands from the computer and send them to the motion slide and the spectrometer. These commands control the automatic movement of the motion slide and the spectral measurement of the spectrometer. By this method, we can measure the optical path of the object measured in different holes on the plate through displacement. In the computer software part, a customized LabVIEW program serves as a control interface for the spectrometer and the motion side, presenting the real-time full spectral absorbance measured by the spectrometer, and the data can be saved to the local excel. With subsequent data processing, the optimal colorimetric wavelength of the measured substance can be determined quickly. These three parts worked together to achieve fast spectral measurements of the solution absorbance in the multi-well plate. In the experiment, we applied the AHTEMR platform to measure the spectra of Bovine Serum Albumin (BSA) and the glucose solution at different concentrations to demonstrate the high-throughput, full-spectrum, and rapid-speed characteristics of the platform. The AHTEMR platform provides a promising approach that enables a fast measurement of the full spectrum of biochemical substances in a high-throughput microplate, which will be useful in biochemical assays for some special substances such as oxidizable substances.

## 2. Materials and Methods

### 2.1. Experimental Setup

**Figure 2:**
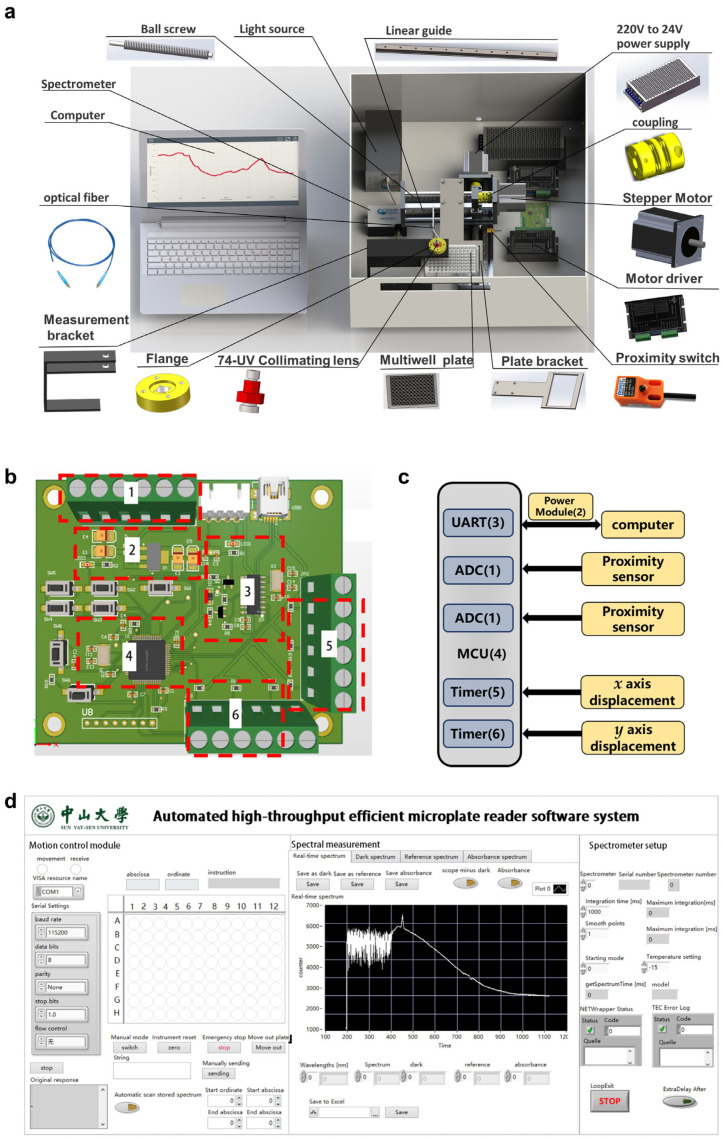
Configuration of the proposed AHTEMR platform: (**a**) Schematic diagram of the AHTEMR. (**b**) Schematic diagram of the main control circuit. (**c**) Block diagram of the control circuit system. (**d**) Computer software operation interface.

A schematic representation of the proposed AHTEMR platform configuration is described in Figure 2a. These components are: shell, light source, 24 V~220 V power supply, ball screw, stepper motor, motor driver, control circuit, proximity switch, plate bracket, 96-well cell culture plate, collimating lens, flange, spectrometer, and computer. The light source (DH-mini, Ocean Optics, Orlando, FL, USA) is a compact deuterium halide light source with a power output in the UV, visible, and NIR range (200~2500 nm). The miniature spectrometer (USB2000 UV-VIS-ES, Ocean Optics, Orlando, FL, USA) covers the wavelength range from 200 to 850 nm with a resolution of 0.399 nm. Two 74-UV collimating lenses (200~2000 nm) are attached to both incident and outgoing edges of the sample for collimation. To achieve precise two-dimensional motion in the *x* and *y* directions of the mechanical system, ball screws are used in conjunction with linear guides and slides. Both sides of the ball screws are attached to the base bearings, and one side is connected to the motor shaft with a coupling to form a one-way motion system. Two-wire track rails with strokes of 100 mm and 200 mm are combined to form a displacement system on the *xy* plane, which is connected to the custom-designed housing by screws. An optical path holder fabricated by welding and milling has been designed to replace the traditional cuvette holder. A flange connects the collimator and the holder for easy replacement and maintenance. The holder is designed and milled to fit the dimensions of the microplate to form a clear light path. One side of the holder is screwed to the top of the motion platform. The housing and the individual parts are painted in matt black color to minimize the interference caused by reflections.

### 2.2. Structure of Control Circuit System

The control circuit system consists of the device, hardware, and software systems. The hardware system includes an analog-to-digital converter, a power supply module, a serial communication module, a microcontroller, and a dual PWM output timer, with red dashed boxes indicating the location of the integrated circuit components. Figure 2b depicts the main control circuit diagram, where (1) is an analog-to-digital conversion interface used to detect proximity switching voltage; (2) is the power module that converts voltage from 5 to 3.3 V; (3) is a serial port communication module with the CH340 chip working as the core, achieving two-way communication with the host computer; (4) is the MCU module with STM32f103RCT6 as the core; and (5) and (6) are motor-driven outputs. The connections between the various modules are shown in Figure 2c. The core of the control circuit is an STMicroelectronics STM32f103RCT6 microcontroller, which receives specific control commands from the host computer through the Universal Asynchronous Receiver/Transmitter (UART) interface and is integrated into the PC via the USB hub together with the spectrometer. To control the motor travel accurately, the PWM signal is outputted through two advanced timers, and it is set to 5 V by adding a 1 k Ohm resistor to the output port to set the pull-up. Then, the 5 V control signal is converted to 24 V through the motor driver (DM542, Haijiejiachuang Co., Ltd., Beijing, China).

LabVIEW software is implemented in the computer for the motion control of the platform as shown in Figure 2d. The graphical interface facilitates the selection of the target well on the microplate without having to control it through a complicated location by coordinates. The control circuit receives the command and performs calculations based on the current and ordered coordinates to derive the movement of the motion platform at a preset speed. Two proximity switches are installed at the starting positions in the *x* and *y* directions and are connected to the Printed Circuit Board (PCB) by wires, which in turn are detected by the analog-to-digital (AD) module of the microcontroller. When the instrument is powered up or when the user clicks the reset button, it will automatically move to the initial point for the reset operation. This ensures accurate control movement with no movement failure. A secondary development is based on the spectrometer Software Development Kit (SDK) provided by Ocean Optics. The real-time spectra can be displayed on the graphical interface, allowing the user to quickly locate useful bands and save the data locally on a PC for subsequent analysis. Furthermore, algorithms programmed in MATLAB software can be embedded in the console to perform a variety of calculations and analyses in real time.

### 2.3. Sample Preparation and Spectral Measurement

**Figure 3:**
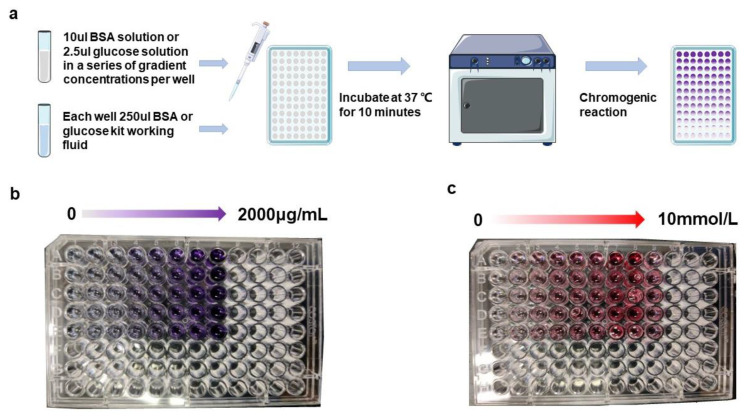
Experimental process of the AHTEMR: (**a**) Flow chart of the BSA color reaction experiment. (**b**) Microplate carrying BSA solution after color development. (**c**) Microplate carrying glucose solution after color development.

As shown in Figure 3a, 1% BSA solution (Source Leaf Bio) was diluted to 2000 μg/mL, 1000 μg/mL, 500 μg/mL, 250 μg/mL, 100 μg/mL, and 50 μg/mL with distilled water. Then, 10 μL of the sample was tested, and 250 μL of the working solution was added dropwise to each well using a pipette gun; the working solution was obtained from a total protein assay kit (A045-4-1, Jiancheng Bioengineering Co., Nanjing, China). Similarly, the glucose solution was diluted to 10 mmol/L, 8 mmol/L, 6 mmol/L, 4 mmol/L, and 2 mmol/L using distilled water. Then, 2.5 μL of the sample was tested, and 250 μL of the working solution was added dropwise to each well using a pipette gun; the working solution was obtained from the kit. Five sets of replicate experiments were set up. After the drops were added, the solution was incubated for 30 min at 37 °C on a thermostat. As shown in Figure 3b,c, the BSA and glucose solution changed from colorless to purple and red color, respectively.

After the prepared samples were placed on the optical path holder, the computer was turned and the spectrum measurement range was set to 200~850 nm. With the setting of stepper motor parameters (a stepper angle of 1.8 degrees) and initialization to zero point (*x* = 0, *y* = 0), the absorbance spectrum after color development was measured and processed in the LabVIEW program. The real-time spectra were therefore displayed on the graphical interface and the useful bands were saved on a PC for subsequent analysis. As the control, the same species and samples with the same concentration measured in AHTEMR were reused in the commercial microplate reader (Infinite M200 PRO, TECAN, Männedorf, Switzerland).

## 3. Results

### 3.1. Experimental Results of the AHTEMR Platform

**Figure 4:**
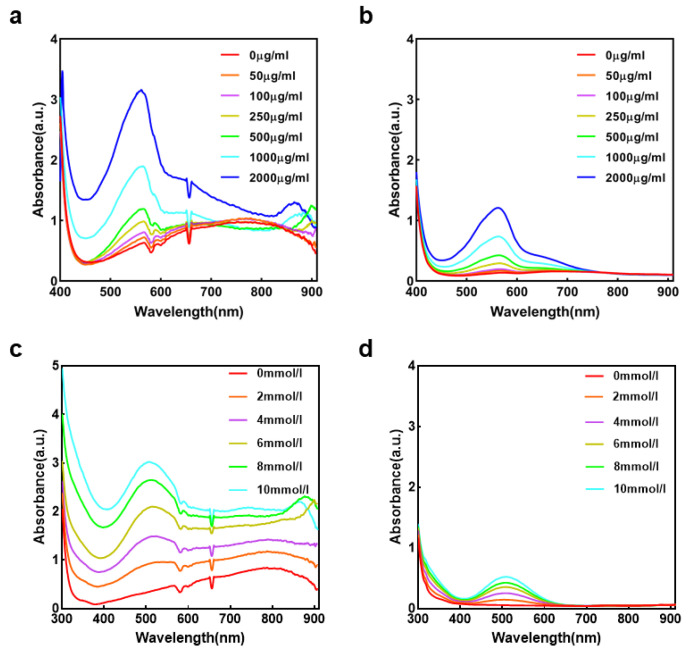
Spectral measurement using the AHTEMR and CMR: (**a**) Absorbance spectrum of BSA at different concentrations measured by the AHTEMR. (**b**) Absorbance spectrum of BSA at different concentrations measured by the CMR. (**c**) Absorbance spectrum of glucose at different concentrations measured by the AHTEMR. (**d**) Absorbance spectrum of glucose at different concentrations measured by the CMR.

To evaluate the performance of the AHTEMR platform, we carried out comparative experiments using the AHTEMR platform and CMR for full-spectrum measurements of BSA and glucose. Results of the BSA measurement are shown in Figure 4a,b. The spectral waveforms measured by the AHTEMR platform range from 400 to 900 nm and are generally consistent with the results measured by the CMR for the BSA. Based on the peak locations in the measured spectrums, it is suggested that the effective wavelengths of the spectral measurements of both the AHTEMR platform and CMR range from 500 to 700 nm, which coincides with the spectra of BSA obtained by reported articles [30]. Results of the glucose measurement are shown in Figure 4c,d. The AHTEMR platform and CMR have a similar absorbance spectrum for the glucose, which is also consistent with the public data [31]. Compared with the result of the BSA measurement, the result of the glucose measurement has a blue-shifted effective wavelength range from 400 to 600 nm due to the optical absorbance property of glucose. Although the AHTEMR realizes the same function, there are still some differences between the AHTEMR and CMR (as shown in Figure 4a–d). Firstly, there are two extra downward peaks around 580 and 660 nm, respectively, in the measured spectrum of the AHTEMR, while there are no peaks in the measured spectrum of the CMR. Secondly, the curves of BSA and glucose obtained by both are not flat when the values of concentration are zero. These two differences between the spectrum measured by the AHTEMR and CMR may be caused by our customized calculation of the absorption spectrum. According to the Beer–Lambert Law, we calculate the absorbance as Equation (1):

A = lg(I_0_/I_X_) (1)

where A is the absorbance, I_0_ is the reference spectrum and I_X_ is the measured spectrum. Thus, the measured spectrum shown in the plotting is with reference to air and contains not only the absorbance of the solution but also the absorbance of the orifice plate.

In both BSA and glucose measurement, spectral absorbance values measured by the AHTEMR platform are about three times that of the CMR. The larger absorbance values will give a more accurate spectrum for the BSA and glucose measurement. Thus, the results demonstrate that our AHTEMR platform is more accurate in measuring the spectrums of biochemical compounds and is able to distinguish different compounds.

To explore the capacity of the AHTEMR platform in distinguishing different concentrations of biochemical compounds, we further carried out comparative experiments using the AHTEMR platform and CMR for full-spectrum measurements of the BSA and glucose solution at different concentrations. For both BSA and glucose measurements, the absorbance spectrums measured by the AHTEMR platform and CMR both increase with the concentration in effective wavelength ranges (from 500 to 700 nm for the BSA and from 400 to 600 nm for the glucose; Figure 4). Particularly, the increases in absorbance measured around the peaks by the AHTEMR platform are much larger, suggesting the ability of peak absorbance values to represent the concentrations of the BSA and glucose solution. The further comparison of the absorbance increases in spectrums between the AHTEMR platform and CMR shows that the absorbance increases measured by the AHTEMR platform are also about three times that of the CMR when the concentrations of BSA increase from 0 to 2000 μg/mL and glucose increase from 0 to 10 mM. These larger absorbance increases are attributed to a larger spectral absorbance value measured by the AHTEMR platform and allow a higher sensitivity for the AHTEMR platform in the BSA and glucose measurement. These results of BSA and glucose concentration measurements have demonstrated that the AHTEMR platform not only can provide a good observation of absorbance spectrum from biochemical compounds just like the CMR, but also have a greater spectral response to the concentration change in biochemical compounds.

### 3.2. The BSA Quantification Using the AHTEMR Platform

**Figure 5:**
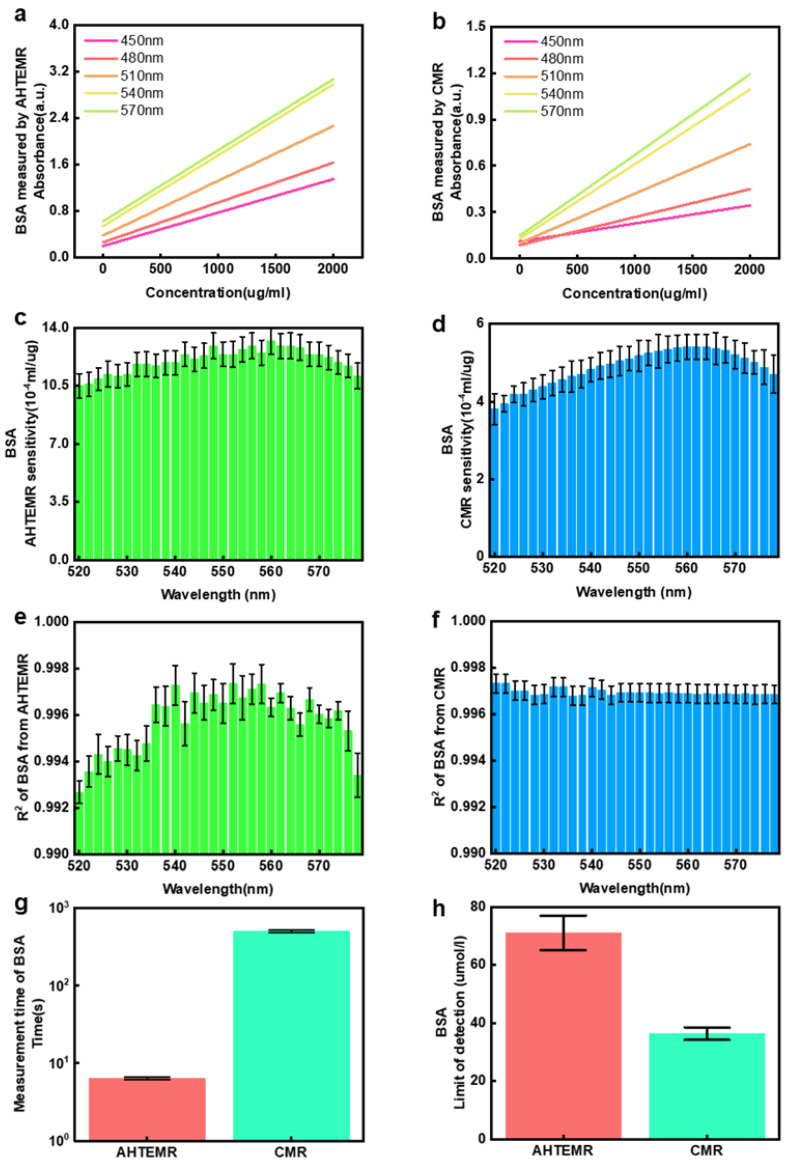
BSA spectral measurement using the AHTEMR and CMR: (**a**) Absorbance spectrum linear fitting results of BSA at different wavelengths measured by the AHTEMR. (**b**) Absorbance spectrum linear fitting results of BSA at different wavelengths measured by the CMR. (**c**) Sensitivity distribution of linear fitting results between 520 and 580 nm measured by the AHTEMR. (**d**) Sensitivity distribution of linear fitting results between 520 and 580 nm measured by the CMR. (**e**) R^2^ distribution of linear fitting results between 520 and 580 nm measured by the AHTEMR. (**f**) R^2^ distribution of linear fitting results between 520 and 580 nm measured by the CMR. (**g**) AHTEMR and CMR measurements of the time spent on the spectral measurement of BSA solution per well. Among them, the AHTEMR costs about 6.37 s per well and the CMR costs about 504.29 s per well. (**h**) Detection limits of the AHTEMR platform and CMR for BSA measurement by 3δ calculation; the detection limit of the AHTEMR is 71.08 μg/mL, and the detection limit of the CMR is 36.34 μg/mL. All experiments were repeated in 3 well with error bars of mean ± SD.

The capacity of the AHTEMR platform for quantifying biochemical compounds at a fixed wavelength was studied by comparing absorbance responses of the AHTEMR platform and CMR to BSA at different spectral wavelengths. The linear fittings of absorbances at different spectral wavelengths for the BSA measured by the AHTEMR platform and CMR are shown in Figure 5a,b, respectively. The absorbances at different spectral wavelengths increase with the concentrations of BSA when measured by both the AHTEMR platform and CMR. The AHTEMR platform and CMR measurements have a similar trend in response to the change in BSA concentration but they show different slopes for the linear fittings, implying that the AHTEMR platform and CMR measurements have different sensitivities at different wavelengths. To find the optimal wavelength of the measurement with the highest sensitivity, we calculated the slopes of the linear fittings of the BSA measurements at different spectral wavelengths. The sensitivity distributions of the linear fittings obtained from the AHTEMR platform and CMR measurements are shown in Figure 5c,d, respectively. The sensitivities of the AHTEMR and CMR are similar in distributions and the maximum sensitivities are both around 562 nm. The wavelength with the maximum sensitivity is consistent with the recommended wavelength for the BSA measurement in the instruction. We also find that the sensitivity of AHTEMR is much larger than that of the CMR and the maximal sensitivity of the AHTEMR at around 562 nm is about 2.38 times the maximal sensitivity of the CMR. This demonstrates that the AHTEMR platform has a better performance in sensitivity when compared to the CMR.

The R-squared values of the linear fittings of the BSA measurement at different spectral wavelengths were calculated to evaluate the linearity of responses of the AHTEMR platform and CMR to the change in BSA concentrations at different wavelengths. Sensitivity distributions of the linear fittings measured by the AHTEMR platform and CMR are shown in Figure 5e,f, respectively. The R-squared values calculated from the AHTEMR platform and CMR measurement show different distributions. The distribution of the AHTEMR platform has a high R-squared value around 0.998 in the wavelength range from 550 to 570 nm and a low R-squared value around 0.994 in the wavelength range from 520 to 540 nm. In contrast, the distribution of the CMR is flat and stays around 0.997 in the wavelength range from 520 to 570 nm. Thus, the linearity of absorbance response of the AHTEMR in the BSA measurement is worse than that of the CMR in the wavelength range from 520 to 540 nm, but it is better than that of the CMR in the wavelength range from 550 to 570 nm. Considering that both the AHTEMR platform and CMR show maximal sensitivity around 560 nm, this may work at a wavelength of 560 nm in practical application. Hence, the linearity of absorbance response of the AHTEMR is better than that of the CMR when using a fixed wavelength of 560 nm in practical application.

Apart from the sensitivity and linearity of responses of the AHTEMR platform and CMR to the change in BSA concentrations at different wavelengths, we also quantified the measurement time that the AHTEMR and CMR cost for full-spectrum measurements to evaluate the measurement speed of both instruments. The statistics regarding the time cost of the AHTEMR and CMR measurements are shown in Figure 5g. The AHTEMR platform costs an average of 6.37 ± 0.23 s per well to obtain a full spectrum of BSA solution, while the CMR costs an average of 504.29 ± 20.17 s per well to obtain a full spectrum of BSA solution. The time cost of the CMR is about 79 times that of the AHTEMR platform for full-spectrum measurement, which demonstrates a higher efficiency of the AHTEMR platform for full-spectrum measurement. We also quantified the detection limits of the AHTEMR platform and CMR for the BSA measurement by a 3δ calculation (Figure 5h). The detection limit of the AHTEMR measurement is 71.08 ± 5.98 μg/mL and the detection limit of the CMR measurement is 36.34 ± 2.14 μg/mL. The lower detection limit of the CMR measurement is due to the smaller standard deviation with better repeatability. Overall, the AHTEMR platform performs better on measurement sensitivity and speed but worse on detection limit than the CMR. After weighing the better and worse performances, we conclude that the AHTEMR is a competent instrument for high-throughput spectrum measurement.

### 3.3. Glucose Quantification Using the AHTEMR Platform

**Figure 6:**
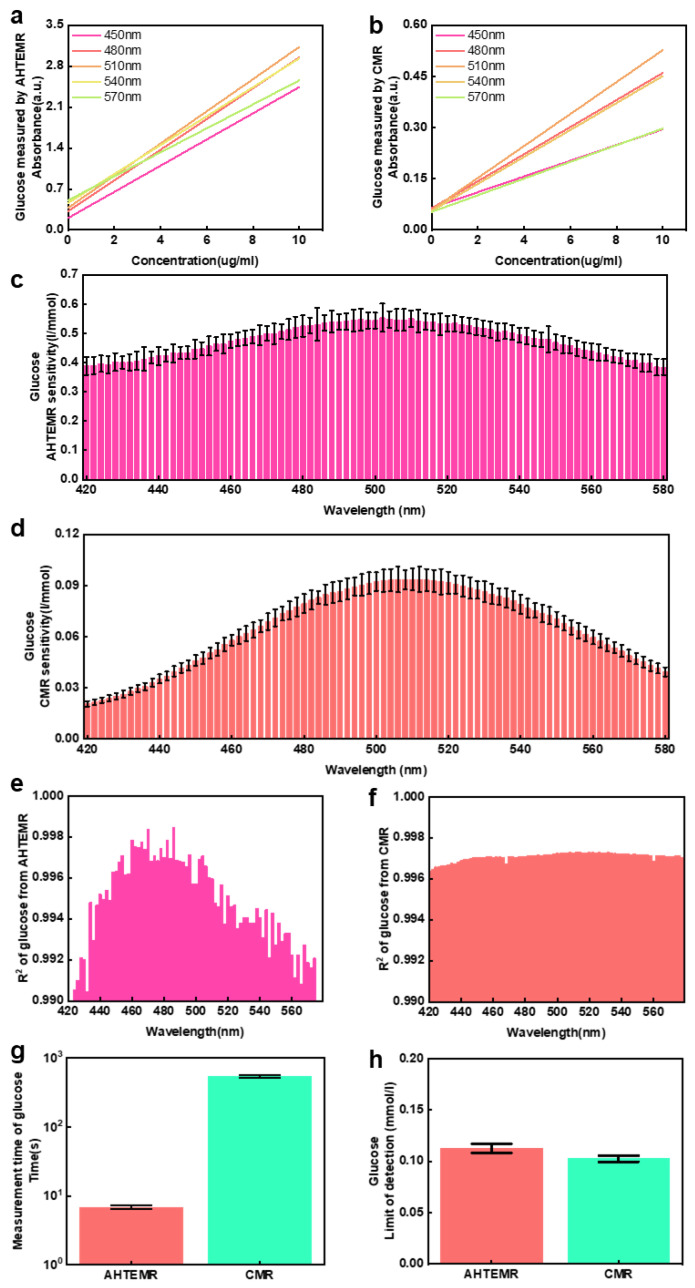
Glucose spectral measurement using the AHTEMR and CMR: (**a**) Absorbance spectrum linear fitting results of glucose at different wavelengths measured by the AHTEMR. (**b**) Absorbance spectrum linear fitting results of glucose at different wavelengths measured by the CMR. (**c**) Sensitivity distribution of linear fitting results between 420 and 580 nm measured by the AHTEMR. (**d**) Sensitivity distribution of linear fitting results between 520 and 580 nm measured by the CMR. (**e**) R^2^ distribution of linear fitting results between 420 and 580 nm measured by the AHTEMR. (**f**) R^2^ distribution of linear fitting results between 420 and 580 nm measured by the CMR. (**g**) AHTEMR and CMR measurements of the time spent on the spectral measurement of glucose solution per well. Between them, the AHTEMR costs about 6.87 s per well and the CMR costs about 536.6 s per well. (**h**) Detection limits of the AHTEMR platform and CMR for glucose measurement by a 3δ calculation; the detection limit of the AHTEMR is 0.1126 mmol/L, while the detection limit of the CMR is 0.1027 mmol/L. All experiments are repeated in 3 well with error bars of mean ± SD.

The capacity of the AHTEMR platform for quantifying biochemical compounds at a fixed wavelength was studied by comparing absorbance responses of the AHTEMR platform and CMR to the glucose solution at different spectral wavelengths. The linear fittings of absorbance at different spectral wavelengths for the glucose by the AHTEMR platform and CMR are shown in Figure 6a,b, respectively. Both the AHTEMR and CMR platforms showed absorbance increasing with the increase in concentrations of the glucose solution at different spectral wavelengths. They presented a similar trend in response to the change in glucose concentrations but showed different slopes of the linear fittings at different wavelengths, which demonstrated different sensitivities for the AHTEMR platform and CMR measurement at different wavelengths. We calculated the slopes of the linear fittings of the glucose measurement at different spectral wavelengths to check the optimal wavelength of the measurement with the maximal sensitivity. Sensitivity distributions of the linear fittings measured by the AHTEMR platform and CMR are shown in Figure 6c,d, respectively. The sensitivity distributions of the AHTEMR and CMR platforms show similar distributions in which the maximal sensitivities of both instruments are around 505 nm. The wavelength showing the maximal sensitivity was consistent with the recommended wavelength for the glucose measurement in the instruction. We also found that the sensitivity of the AHTEMR was much higher than that of the CMR and the maximal sensitivity of the AHTEMR was around 505 nm, which was about 5.84 times that of the CMR. This demonstrates that the AHTEMR platform has a better performance in sensitivity than the CMR.

We calculated the R-squared values of linear fittings of the glucose measurement at different spectral wavelengths to evaluate the linearity of responses of the AHTEMR and the CMR to the change in glucose concentration at different wavelengths. The sensitivity distributions of the linear fittings measured by the AHTEMR platform and CMR are shown in Figure 6e,f, respectively. The R-squared values calculated from the AHTEMR and CMR measurement show different distributions. The distribution from the AHTEMR platform has a high R-squared value around 0.997 with wavelengths ranging from 460 to 520 nm and a low R-squared value around 0.994 with wavelengths ranging from 520 to 580 nm. In contrast, the distribution of the CMR is flat and stays around 0.996 with wavelengths ranging from 420 to 580 nm. Therefore, the linearity of absorbance response of the AHTEMR platform in glucose measurement is worse than that of the CMR in wavelengths ranging from 520 to 580 nm, but it is better than that of the CMR in wavelength ranging from 460 to 520 nm. Given both the AHTEMR platform and CMR showed maximum sensitivity around 505 nm, and may work at a wavelength of 505 nm in practical application, the linearity of absorbance response of the AHTEMR platform could be better than that of the CMR.

In addition to the sensitivity and linearity of response of the AHTEMR platform and CMR to the change in glucose concentrations at different wavelengths, we quantified the time that the AHTEMR and CMR cost for full-spectrum measurement to evaluate the measurement speed of the AHTEMR and CMR. The statistics of the time cost of the AHTEMR platform and CMR measurements are shown in Figure 6g. The AHTEMR platform costs 6.87 ± 0.33 s to obtain the full spectra of glucose solution per well, while the CMR costs 536.60 ± 28.00 s to obtain the full spectra of glucose solution per well. The time cost of the CMR is about 78 times that of the AHTEMR platform for full-spectrum measurement, suggesting a lower efficiency of the CMR in full-spectrum measurements. We also quantified the detection limits of the AHTEMR platform and CMR for glucose measurement by a 3δ calculation (Figure 6h). The detection limit of the AHTEMR measurement is 0.11264 ± 0.00448 mmol/L and the detection limit of the CMR measurement is 0.10266 ± 0.00308 mmol/L. The detection limit of the AHTEMR is close to that of the CMR, and to some extent, the AHTEMR has reached the commercial test performance. Compared with BSA, the results of measuring glucose with the AHTEMR are better. Overall, compared with the CMR, the AHTEMR performs better in terms of sensitivity and speed, but is similar in terms of the detection limit. After weighing the better and worse performances, the AHTEMR is a comparable instrument for high-throughput spectroscopic measurements.

## 4. Discussion

Our work showed an improved AHTEMR platform for a fast and high-throughput measurement of the full spectrum of biochemical compounds. Compared with the commercial instruments, our AHTEMR platform has two advantages. The first advantage of our AHTEMR platform is the high speed of spectral measurement for biochemical compounds. The time cost of the AHTEMR platform to measure the full spectrum of biochemical solution per sample is less than ten seconds, whereas the time cost of a commercial instrument to measure the full spectrum of biochemical solution per sample is about five hundred seconds. Even compared with the ThermoScientific Multiskan SkyHigh Microplate, which is declared the fastest microplate reader, our ATHEMR platform can save about five hundred seconds in one single spectral measurement of BSA and glucose solution. Considering that the 24-well plate was a commonly used carrier in a batch of experiments, the usage of the AHTEMR platform can save several hours for a batch of experiments measuring the spectra of biochemical compounds. The second advantage is the large intensity of the absorbance spectral measurement for biochemical compounds. The spectral absorbance values measured by the AHTEMR platform are about three times that of the commercial instrument at different wavelengths of the spectrum. The larger intensity of absorbance spectrum demonstrated a higher sensitivity of the AHTEMR platform when compared with that of the commercial instrument. The sensitivities of the AHTEMR platform are about 2.38 times and 5.84 times that of the commercial instrument in measurements of BSA and glucose solution, respectively. Overall, our AHTEMR performs better in terms of speed and sensitivity for high-throughput spectral measurement.

Despite the advantages of the AHTEMR platform, the performance of this platform in detection limits is worse than that of the commercial instrument. The detection limits of the AHTEMR platform were 71.08 ± 5.98 μg/mL and 0.11264 ± 0.00448 mmol/L for the measurement of BSA and glucose solution, respectively, whereas the detection limits of the commercial instrument were 36.34 ± 2.14 μg/mL and 0.10266 ± 0.00308 mmol/L for the measurement of BSA and glucose solution, respectively. The larger detection limits of the AHTEMR platform may result from the large noise of spectral measurements using the AHTEMR platform, which gives rise to large standard deviations in the 3δ calculation of the detection limit. More specifically, different measuring principles of the AHTEMR and CMR give rise to different detection limits and measuring speeds between the AHTEMR and CMR. The CMR uses a photomultiplier tube to receive the monochromatic light at different wavelengths to measure the full spectrum of biochemical compounds. The accuracy of absorption measurement at a single wavelength is high but the speed of the measurement is low because of the serial detection for each wavelength. In contrast, the AHTEMR uses a grating spectrometer to measure spectral information at different wavelengths within several hundreds of wavelength ranges in parallel. Thus, the speed of the AHTEMR measurement is high, but the accuracy of the AHTEMR measurement at a single wavelength is low. Our AHTEMR platform achieves a rapid spectral measurement by taking advantage of a grating spectrometer that obtains the full spectrum each time without switching monochromatic light.

To further improve the performance of the AHTEMR platform in the detection limit, we will reduce the noise of spectral measurement using the AHTEMR platform in two ways in future work. The first method is to increase the integral time of the spectral measurement using the AHTEMR platform, which will reduce the random noise in the measurements by integrating more spectral recordings together. Although the increase in integral time will make the spectral measurement slower, the improvement of the detection limit enables the AHTEMR platform to achieve a better balance on the overall performance. The second method is to design a digital filter in signal processing to reduce the noise of the AHTEMR measurement. The digital filter will remove the noise from the spectral signal, which may potentially shift the spectrum in wavelength. Further work is needed to explore how to remove the noise from the signal without a large spectral shift.

## 5. Conclusions

In this work, we have developed an AHTEMR platform for the fast and high-throughput measurement of the full spectrum of biochemical compounds. The AHTEMR platform can accurately measure the spectrum of biochemical compounds ranging from 300 to 900 nm for the BSA and glucose solution. The measuring speed of the AHTEMR platform is 100 times faster than that of the CMR and the sensitivity of the AHTEMR platform to concentration change is about three times higher than that of the CMR. Although the detection limit of the AHTEMR platform is slightly worse than that of the CMR in the BSA and glucose measurement, the AHTEMR platform is still a competitive tool for spectrum measurements considering its speed and sensitivity. Our AHTEMR platform can serve as a high-throughput microplate reader platform for measuring the full spectrum of biochemical compounds with great efficiency.

## Data Availability

Datasets generated during and/or analyzed during the current study are available from the corresponding author on reasonable request.

## References

[1] Zhang Y., Chen M., Liu C., Chen J., Luo X., Xue Y., Liang Q., Zhou L., Tao Y., Li M. (2021). Sensitive and rapid on-site detection of SARS-CoV-2 using a gold nanoparticle-based high-throughput platform coupled with CRISPR/Cas12-assisted RT-LAMP. Sens. Actuators B Chem..

[2] Chaianantakul N., Wutthi K., Kamput N., Pramanpol N., Janphuang P., Pummara W., Phimon K., Phatthanakun R. (2018). Development of mini-spectrophotometer for determination of plasma glucose. Spectrochim. Acta A Mol. Biomol. Spectrosc..

[3] Freeman M.J., Sands-Freeman L., Armstrong C.H. (1983). Comparison of manually performed Microtiter plate and semiautomated (cuvette) indirect enzyme-linked immunosorbent assays for the serodiagnosis of mycoplasmal pneumonia of swine. Am. J. Vet. Res..

[4] Platton S., Bartlett A., MacCallum P., Makris M., McDonald V., Singh D., Scully M., Pavord S. (2021). Evaluation of laboratory assays for anti-platelet factor 4 antibodies after ChAdOx1 nCOV-19 vaccination. J. Thromb. Haemost..

[5] Li Y., Deng F., Hall T., Vesey G., Goldys E.M. (2021). CRISPR/Cas12a-powered immunosensor suitable for ultra-sensitive whole Cryptosporidium oocyst detection from water samples using a plate reader. Water Res..

[6] Sharma P., Kumar S. (2021). Bioremediation of heavy metals from industrial effluents by endophytes and their metabolic activity: Recent advances. Bioresour. Technol..

[7] Matschulat D., Deng A., Niessner R., Knopp D. (2005). Development of a highly sensitive monoclonal antibody based ELISA for detection of benzo[a]pyrene in potable water. Analyst.

[8] Carr A.C., Bozonet S., Pullar J., Spencer E., Rosengrave P., Shaw G. (2021). Neutrophils isolated from septic patients exhibit elevated uptake of vitamin C and normal intracellular concentrations despite a low vitamin C Milieu. Antioxidants.

[9] Huang L., Ding L., Zhou J., Chen S., Chen F., Zhao C., Xu J., Hu W., Ji J., Xu H. (2021). One-step rapid quantification of SARS-CoV-2 virus particles via low-cost nanoplasmonic sensors in generic microplate reader and point-of-care device. Biosens. Bioelectron..

[10] Zhang J., Wu J., Li H., Chen Q., Lin J.M. (2015). An in vitro liver model on microfluidic device for analysis of capecitabine metabolite using mass spectrometer as detector. Biosens. Bioelectron..

[11] Xi Z., Huang R., Li Z., He N., Wang T., Su E., Deng Y. (2015). Selection of HBsAg-specific DNA aptamers based on carboxylated magnetic nanoparticles and their application in the rapid and simple detection of hepatitis B virus infection. ACS Appl. Mater. Interfaces.

[12] Song Z., Lin T., Lin L., Lin S., Fu F., Wang X., Guo L. (2016). Invisible security ink based on water-soluble graphitic carbon nitride quantum dots. Angew. Chem. Int. Ed. Engl..

[13] Mattsson N., Andreasson U., Persson S., Carrillo M.C., Collins S., Chalbot S., Cutler N., Dufour-Rainfray D., Fagan A.M., Heegaard N.H. (2013). CSF biomarker variability in the Alzheimer’s Association quality control program. Alzheimers Dement..

[14] Toh S.Y., Citartan M., Gopinath S.C., Tang T.H. (2015). Aptamers as a replacement for antibodies in enzyme-linked immunosorbent assay. Biosens. Bioelectron..

[15] Liu D., Ju C., Han C., Shi R., Chen X., Duan D., Yan J., Yan X. (2020). Nanozyme chemiluminescence paper test for rapid and sensitive detection of SARS-CoV-2 antigen. Biosens. Bioelectron..

[16] Hu J., Wang S., Wang L., Li F., Pingguan-Murphy B., Lu T.J., Xu F. (2014). Advances in paper-based point-of-care diagnostics. Biosens. Bioelectron..

[17] Raouia A., Aziz A. (2022). Highly selective and sensitive detection of cadmium ions by horseradish peroxidase enzyme inhibition using a colorimetric microplate reader and smartphone paper-based analytical device. Microchem. J..

[18] Su K., Zou Q., Zhou J., Zou L., Li H., Wang T., Hu N., Wang P. (2015). High-sensitive and high-efficient biochemical analysis method using a bionic electronic eye in combination with a smartphone-based colorimetric reader system. Sens. Actuators B Chem..

[19] MacIsaac S.A., Sweeney C.L., Gagnon G.A. (2021). Instrument hacking: Repurposing and recoding a multiwell instrument for automated, high-throughput monochromatic UV photooxidation of organic compounds. ACS ES&T Eng..

[20] Loftin K.A., Graham J.L., Hilborn E.D., Lehmann S.C., Meyer M.T., Dietze J.E., Griffith C.B. (2016). Cyanotoxins in inland lakes of the United States: Occurrence and potential recreational health risks in the EPA National Lakes Assessment 2007. Harmful Algae.

[21] Avrutsky I., Chaganti K., Salakhutdinov I., Auner G. (2006). Concept of a miniature optical spectrometer using integrated optical and micro-optical components. Appl. Opt..

[22] Kim S., Lee Y., Kim J.Y., Yang J.H., Kwon H.J., Hwang J.Y., Moon C., Jang J.E. (2019). Color-sensitive and spectrometer-free plasmonic sensor for biosensing applications. Biosens. Bioelectron..

[23] Choi J., Lee J., Jung J.H. (2020). Fully integrated optofluidic SERS platform for real-time and continuous characterization of airborne microorganisms. Biosens. Bioelectron..

[24] Wang L.J., Chang Y.C., Sun R., Li L. (2017). A multichannel smartphone optical biosensor for high-throughput point-of-care diagnostics. Biosens. Bioelectron..

[25] Hassanain W.A., Izake E.L., Schmidt M.S., Ayoko G.A. (2017). Gold nanomaterials for the selective capturing and SERS diagnosis of toxins in aqueous and biological fluids. Biosens. Bioelectron..

[26] Zhang C., Lai C., Zeng G., Huang D., Tang L., Yang C., Zhou Y., Qin L., Cheng M. (2016). Nanoporous Au-based chronocoulometric aptasensor for amplified detection of Pb(2+) using DNAzyme modified with Au nanoparticles. Biosens. Bioelectron..

[27] Butler H.J., Ashton L., Bird B., Cinque G., Curtis K., Dorney J., Esmonde-White K., Fullwood N.J., Gardner B., Martin-Hirsch P.L. (2016). Using Raman spectroscopy to characterize biological materials. Nat. Protoc..

[28] Pan C., Zhu B., Yu C. (2020). A dual immunological raman-enabled crosschecking test (DIRECT) for detection of bacteria in low moisture food. Biosensors.

[29] Zhu W., Wen B.Y., Jie L.J., Tian X.D., Yang Z.L., Radjenovic P.M., Luo S.Y., Tian Z.Q., Li J.F. (2020). Rapid and low-cost quantitative detection of creatinine in human urine with a portable Raman spectrometer. Biosens. Bioelectron..

[30] Chen L., Yu Z., Lee Y., Wang X., Zhao B., Jung Y.M. (2012). Quantitative evaluation of proteins with bicinchoninic acid (BCA): Resonance Raman and surface-enhanced resonance Raman scattering-based methods. Analyst.

[31] Yang S.J., Liu D.L., Meng Q.B., Wu S.Y., Song X.M. (2018). Reduced graphene oxide-supported methylene blue nanocomposite as a glucose oxidase-mimetic for electrochemical glucose sensing. RSC Adv..

